# Studies on the Alkali–Silica Reaction Rim in a Simplified Calcium–Alkali–Silicate System

**DOI:** 10.3390/ma9080670

**Published:** 2016-08-09

**Authors:** Kunpeng Zheng, Peter Adriaensens, Geert De Schutter, Guang Ye, Luc Taerwe

**Affiliations:** 1 Magnel Laboratory for Concrete Research, Department of Structural Engineering, Ghent University, Ghent 9000, Belgium; geert.deschutter@ugent.be (G.D.S.); G.Ye@tudelft.nl (G.Y.); Luc.Taerwe@UGent.be (L.T.); 2 Applied and Analytical Chemistry, Institute for Materials Research, University of Hasselt, Hasselt 3590, Belgium; peter.adriaensens@uhasselt.be; 3 Microlab, Faculty of Civil Engineering and Geosciences, Delft University of Technology, Delft 2628, The Netherlands

**Keywords:** alkali–silica reaction, reaction rim, calcium alkali silicate, transport barrier

## Abstract

This work is intended to provide a better understanding about the properties and roles of the reaction rim in an alkali–silica reaction. A simplified calcium–alkali–silicate system was created to simulate the multiple interactions among reactive silica, alkaline solution and portlandite near the aggregate surface during the formation and evolution of the reaction rim in an alkali–silica reaction. A transport barrier preventing the migration of calcium and silicate through itself was found on the interface between the alkali silicate and the calcium hydroxide. The barrier was mainly composed of calcium alkali silicate with silicon–oxygen organizations of Q^2^ and Q^3^ according to the results of ^29^Si nuclear magnetic resonance, the calcium to silica mole ratio ranging from 0.22 to 0.53 and the alkali to silica ratio ranging from 0.20 to 0.26 based the location of the elemental compositional analysis and the storage period of the system.

## 1. Introduction

Alkali–silica reaction (ASR) is one of the most common and serious durability problems of concrete. The mechanism of the generation and accumulation of the interior expansive force at the micro scale leading to expansion and cracking at the macro scale is one of the most critical and controversial issues of ASR.

Many theories and models have been proposed to provide illuminative information to investigate this mechanism [1,2,3,4,5]. During this exploration period over decades, an uncommon phenomenon occurring with ASR, i.e., formation of a “reaction rim”, has drawn our attention. In the earlier studies [6,7,8,9] dealing with the reaction rim, the reaction rim was only considered as one of the by-products of ASR until Ichikawa and Miura in 2007 [10] closely linked the formation of the reaction rim with the generation and accumulation of the interior expansive force.

**Figure 1:**
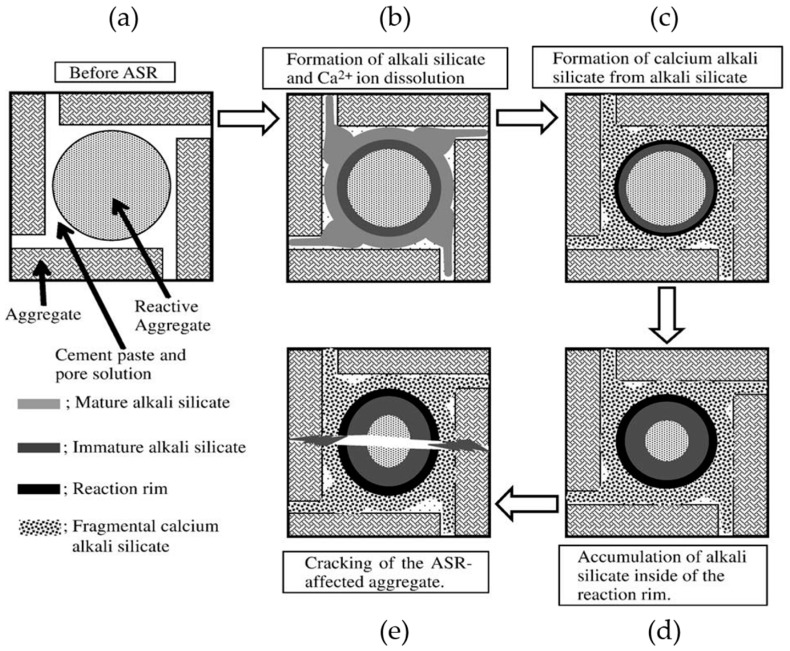
Schematic representation of the mechanism of ASR in concrete modified from [11]: (**a**) before ASR; (**b**) formation of alkali silicate; (**c**) formation of calcium alkali silicate from alkali silicate; (**d**) accumulation of alkali silicate inside of the reaction rim; (**e**) cracking of the ASR-affected aggregate.

**Figure 2:**
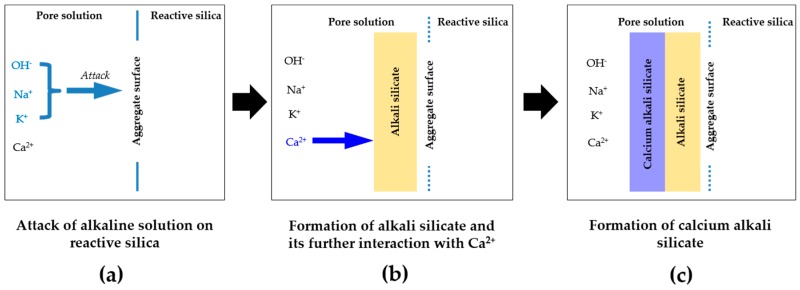
Schematic representation of the interaction of the reactive silica with the constituents in the pore solution of cement paste: (**a**) attack of alkaline solution on reactive silica; (**b**) formation of alkali silicate and its further interaction with Ca^2+^; (**c**) formation of calcium alkali silicate.

Ichikawa’s theory [10,11] is schematically shown in Figure 1 and illustrated as follows. Firstly, alkalis and hydroxyls from the pore solution of cement paste attack the reactive silica in the aggregates to generate alkali silicate, as shown in Figure 1a,b. Secondly, the alkali silicate reacts with calcium from the pore solution of cement paste to form calcium alkali silicate, as shown in Figure 1c. These two steps are illustrated in detail in Figure 2. Thirdly, the reaction rim forms from the formation of calcium alkali silicate. Once the reactive silica particle is completely covered by the reaction rim, the rim can prevent the migration of the alkali silicate from the vicinity of the reactive silica particle to the cement paste. In the meantime, however, the reaction rim allows the penetration of alkaline solution to reach the reactive silica particles resulting in a continuous formation of alkali silicate in the region between the reaction rim and the reactive silica particle. As a consequence, alkali silicate is able to accumulate in that region and generate an expansive force on the reaction rim, which applies a constraint on that region, as shown in Figure 1d. Once this expansive force exceeds the strength of the reaction rim, cracking will occur to break the reaction rim, as shown in Figure 1e.

Unfortunately, why the reaction rim composed of calcium alkali silicate is able to act as a semi-permeable membrane to prevent the penetration of alkali silicate while allowing that of the alkaline solution is not clear from a chemical point of view. Moreover, to what extent the constraint can be provided by the reaction rim until its breakage by the expansive force is unknown as well. In the present study, the emphasis is placed on the former topic.

Obviously, the formation of calcium alkali silicate from the interaction of alkali silicate with calcium is the decisive step for the formation, evolution and operation of the reaction rim. Furthermore, according to Figure 1 and Figure 2, the interface between the reactive silica in aggregate and the pore solution of the cement paste is the very place where the formation and evolution of the reaction rim take place. However, the ambiguousness of the multiple interactions among silica, alkaline solution and calcium as well as the heterogeneous conditions on such an interface makes it extremely difficult to understand the reaction rim from a fundamental chemical point of view.

In light of this consideration, in the present study, a chemical model system was created to simulate the multiple interaction among silica, alkaline solution and calcium at the interface between the reactive silica in aggregates and the pore solution of cement paste during the formation and evolution of the reaction rim in real concrete. With the help of such a model system, the formation of calcium alkali silicate from the interaction between alkali silicate and calcium at their interface was realized to illustrate the formation and evolution of the reaction rim, providing information for a better understanding about the reaction rim and the mechanisms of ASR in real concrete.

## 2. Materials and Methods

### 2.1. Raw Materials

**Figure 3:**
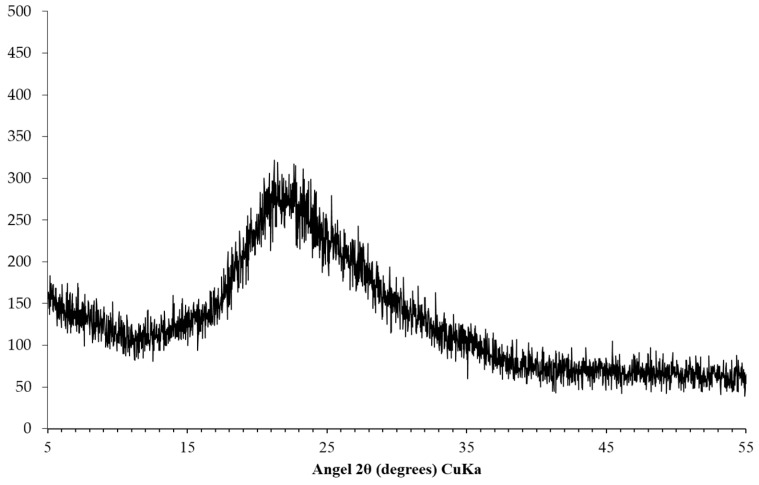
XRD pattern of the silica fume.

In order to simulate the interaction between the reactive silica and the constituents in the pore solution to generate alkali silicate in the first step of the formation of the reaction rim with an accelerated reaction rate, a kind of silica fume (Elekem Microsilica Grade 940U, Oslo, Norway) instead of reactive silica was used in this study. The composition of the silica fume expressed as oxides is given in Table 1 and the X-ray diffraction (XRD, Thermo Scientific ARL X’Tra Diffractometer, Thermo Fisher Scientific Inc., Waltham, MA, USA) pattern of the silica fume is shown in Figure 3. A 1 mol/L NaOH solution was used as the source of alkali and hydroxyl throughout this study. This solution was obtained by dissolving pellets of NaOH in the distilled and CO_2_-free water. The slurry of Ca(OH)_2_ was obtained by adding a certain amount of powdered Ca(OH)_2_ to the 1 mol/L NaOH solution with the liquid to solid mass ratio of 3. The Ca(OH)_2_ slurry was to simulate the calcium source in concrete that, it is present both in the pore solution of the cement paste in the form of Ca^2+^ and in the vicinity of the aggregate surface predominantly in the form of free portlandite. The reason for the addition of NaOH solution during the preparation of Ca(OH)_2_ slurry was to maintain the liquid to solid ratio and the pH level of the whole system at a relatively constant level. The Ca(OH)_2_ slurry was kept mixing on the rotary mixer to maintain its homogeneity until its use. The pellets of NaOH and powder-like Ca(OH)_2_ were both reagent grade.

### 2.2. Preparation of Alkali Silicate and Calcium Alkali Silicate

**Figure 4:**
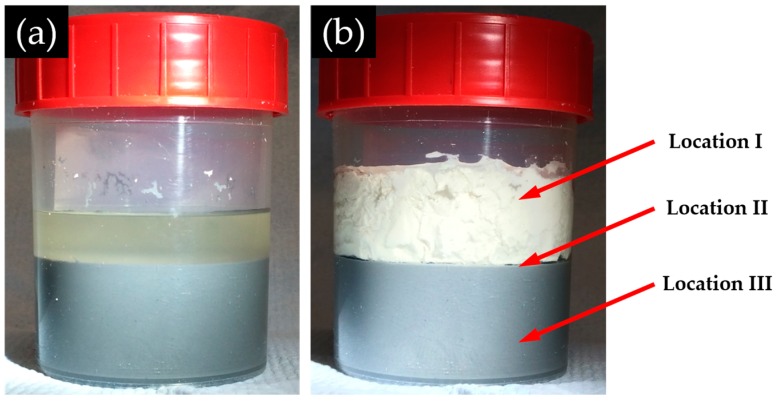
The appearance of the mixture: (**a**) before Ca(OH)_2_ addition; and (**b**) after the addition of Ca(OH)_2_.

The alkali silicate slurry, as the simulated product of the alkaline attack on reactive silica, was prepared by adding the 1 mol/L NaOH solution to the silica fume, sealed in a polypropylene bottle filled with N_2_ and mixed with a rotary mixer at a speed of 60 rpm at room temperature. The liquid to solid mass ratio was set to 3 to simulate the partial silica dissolution in ASR [12]. The mixing was stopped after 24 h. Subsequently, the mixture was left at rest for another 24 h. This allows the stratification of the mixture leading to a stable system with a clear liquid layer at the top and a black slurry layer at the bottom (Figure 4a). This state simulates the situation near the surface of reactive silica during the formation of the reaction rim in ASR where unreacted silica, dissolved and undissolved alkali silicate coexist. After the desired standing period, the Ca(OH)_2_ slurry was added softly to the top of the mixture without any shaking or stirring to avoid disturbance of the layered structure (Figure 4b). The amount of added calcium was set to make the calcium to silica mole (Ca/Si) ratio of the whole system as 0.3 to ensure excess presence of Ca(OH)_2_ near the interface. This step is to simulate the contact of alkali silicate with the calcium source which is present as either Ca^2+^ or Ca(OH)_2_ in the second step of the formation of the reaction rim shown in Figure 2. Afterwards, the mixture was sealed again to avoid contamination and left at rest for another one day (1 D), four days (4 D) and seven days (7 D) to let the reactions continue and the structure evolve before the collection of samples.

### 2.3. Sample Collection and Treatment

**Figure 5:**
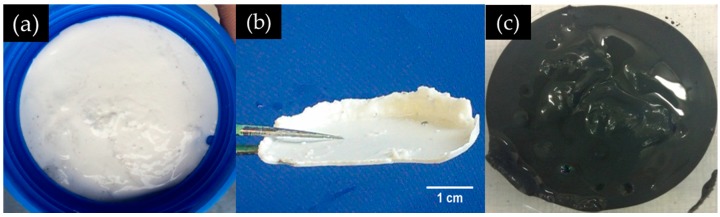
The appearance of the samples from different positions: (**a**) paste-like sample from the middle layer; (**b**) sheet-like sample from the hard layer; and (**c**) slurry-like sample from the slurry layer.

After the desired standing period, paste-like sample (Figure 5a), sheet-like sample (Figure 5b) and slurry-like sample (Figure 5c) were collected from Location I, Location II and Location III, respectively, of the system, as shown in Figure 4. The state of the system when the samples were collected will be introduced in the next section.

**Figure 6:**
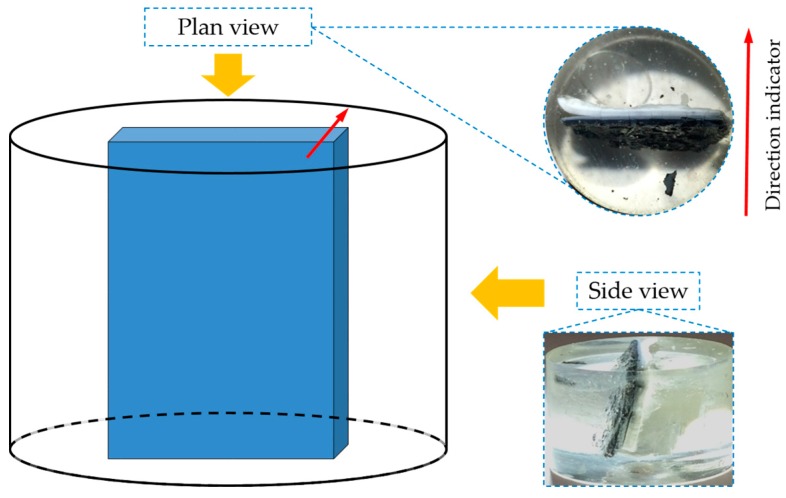
The sheet-like sample fixed vertically in an epoxy.

All the fresh samples were immediately immersed in isopropanol for seven days to arrest further reactions. Isopropanol was renewed every day. After the desired immersion period of seven days, the samples for the powder X-Ray Diffraction (XRD) analysis, the thermogravimetric analysis/differential thermal analysis (TGA/DTA, NETZSCH STA 449 F3 Jupiter thermal analyzer, Netzsch, Selb, Germany) and the X-Ray Fluorescence (XRF, NEX CG, Rigaku, Tokyo, Japan) analysis were taken out of isopropanol and dried with vacuum under room temperature for 2 days (enough for the samples with a small amount). The sheet-like samples for the Environmental Scanning Electron Microscope (ESEM, Philips XL30, Philips, Amsterdam, The Netherlands) equipped with Energy-dispersive X-ray spectroscopy (EDS, EDAM 3 EDS system, EDAX, Tilburg, The Netherlands) measurements were taken out of the isopropanol, dried in a desiccator filled with N_2_ for 2 weeks to avoid any damage during vacuum drying. Portlandite and silica gel were also present in the desiccator for removing any possible CO_2_ and moisture. Thereafter, the sheet-like samples were impregnated in an epoxy platform vertically (see Figure 6) to expose their cross sections. Notably, in order to preserve the original morphology of the sheet-like sample for the ESEM-EDS analysis, the adhesives attached to the sheet-like samples were not removed. Afterwards, the fixed samples were stored in a desiccator filled with N_2_ during the hardening of epoxy for one day. The polishing step was followed according to the procedure proposed by Stuzman [13] to ensure accurate EDS results [14]. For the sheet-like samples, for ^29^Si solid-state cross-polarization (CP)/Magic angle spinning (MAS,) nuclear magnetic resonance (NMR, Agilent Technologies Inc., Santa Clara, CA, USA) analysis, additional steps of sample preparation were followed and described as follows. After the sheet-like sample was collected from the system and immersed in isopropanol, the adhesives attached to the sheet-like sample were removed with a brush. Subsequently, the sample was immersed in isopropanol for seven days to cease further reactions. Afterwards, the sample was dried for 48 h under vacuum. Eventually, the sheet-like sample was crushed and ground into powder. This step was carried out in a glove box filled with N_2_ to avoid contamination.

### 2.4. Characterization Methods

XRD measurements were carried out to identify crystalline phases by using a Thermo Scientific ARL X’Tra Diffractometer equipped with a Peltier cooled detector at a scan rate of 0.8°/min. TGA/DTA were carried out to identify specific phases by quantifying the mass loss at different temperatures. During the measurements of TGA/DTA, the samples were heated from room temperature to 1000 °C at a rate of 10 K/min under N_2_ atmosphere to avoid any contamination.

For the investigations at a micro scale, the samples were examined with backscattered electrons (BSE) detector in ESEM. The elemental composition of the powder-like samples was measured with XRF, the elemental compositions of chosen areas on the hard layers from different samples were measured with EDS equipped with ESEM. ^29^Si CP/MAS NMR spectra were acquired at ambient temperature on a Varian Inova 400 spectrometer (9.4 T wide bore magnet) [15] in 7 mm ceramic zirkonia rotors tightly closed with a double o-ringed Torlon end cap. Magic angle spinning was performed at 4 kHz. Both probe (12 L/min) and upper-barrel (12 L/min) cooling were used to avoid sample heating during the experiments. The signal of talc was used to determine the Hartmann-Hahn condition (ω_1H_ = γ_H_ B_1H_ = γ_Si_ B_1Si_ = ω_1Si_) for cross-polarization and to calibrate the silicon chemical shift scale (−98.0 ppm). Acquisition parameters used were: a spectral width of 28.3 kHz, a 90° pulse of 7.9 μs, a spin-lock field for CP of 40 kHz, a contact time for cross-polarization of 1.5 ms, an acquisition time of 15 ms, a recycle delay time of 5 s and 20,000–40,000 accumulations. High power proton dipolar decoupling during the acquisition time was set to 65 kHz.

## 3. Results and Discussion

### 3.1. System State Description

**Figure 7:**
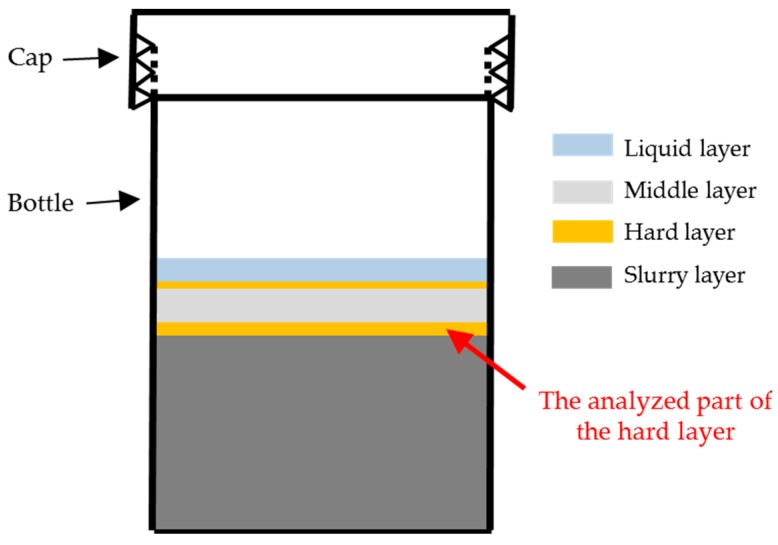
Schematic representation of the layered mixture.

Twenty-four hours after calcium was added, according to the observation during the collection of samples from different places of the system, the appearance of the layered system is schematically shown in Figure 7. At the top, there is a liquid layer. Beneath, the middle layer, where the paste-like sample (Figure 5a) was taken from, is wrapped by a white thin hard layer (the hard layer, where the sheet-like sample (Figure 5b) was taken from) separating the middle layer from the liquid layer above and the slurry layer (where the slurry-like sample (Figure 5c) was taken from) beneath as well.

### 3.2. XRD Results

**Figure 8:**
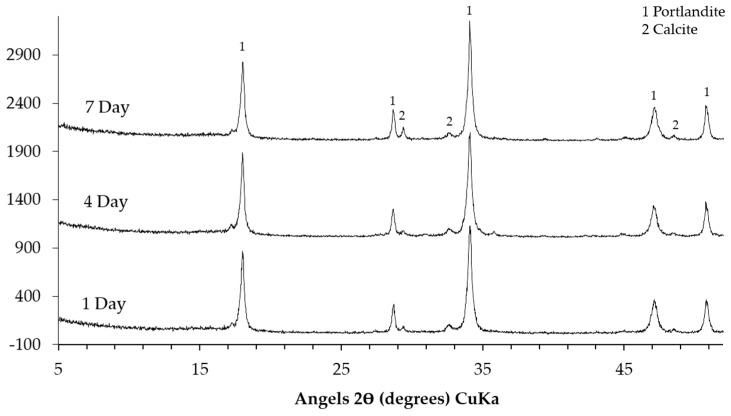
XRD patterns of the sample collected from the middle layer (1, portlandite; 2, calcite).

The XRD patterns of the sample collected from the middle layer are shown in Figure 8. Obviously, the XRD patterns of the sample either from the system reacted for one, four or seven days are similar. This indicates that the different reaction periods did not have significant influence on the kinds of phases in the middle layer. The characteristic peaks at around 18° (2θ), 28.5° (2θ), 34° (2θ), 47° (2θ) and 50° (2θ) confirm the presence of Ca(OH)_2_. The peaks from the calcite as the sign of carbonation in the middle layer are noticed as well. In addition, it seems that calcium alkali silicate was probably not present in the middle layer since its typical broadening and weak peaks were not found.

**Figure 9:**
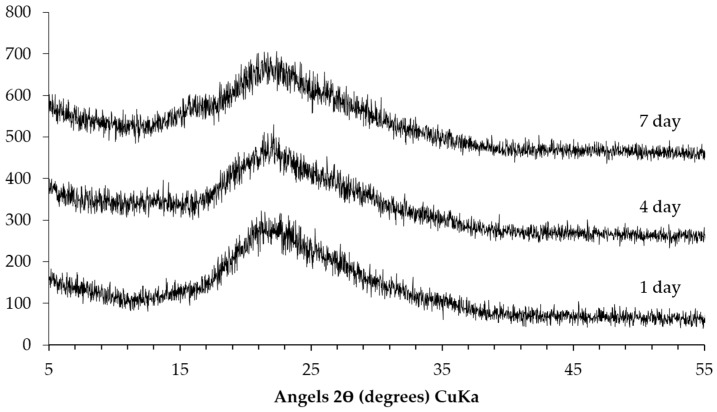
XRD patterns of the sample collected from the slurry layer.

The XRD patterns of the sample collected from the slurry layer are shown in Figure 9. Similar to the trend mentioned in the previous paragraph, the storage period has hardly any influence on the kinds of phases in the slurry layer as well. Particularly, the broadening peak centered around 21° (2θ) indicates the presence of unreacted silica fume or alkali silicate. Moreover, there are no characteristic peaks from Ca(OH)_2_ and calcite indicating the absence of these phases in the slurry layer. The absence of the typical broadening peaks from calcium alkali silicate suggests that there is little or even no calcium alkali silicate present in this layer.

### 3.3. TGA Results

**Figure 10:**
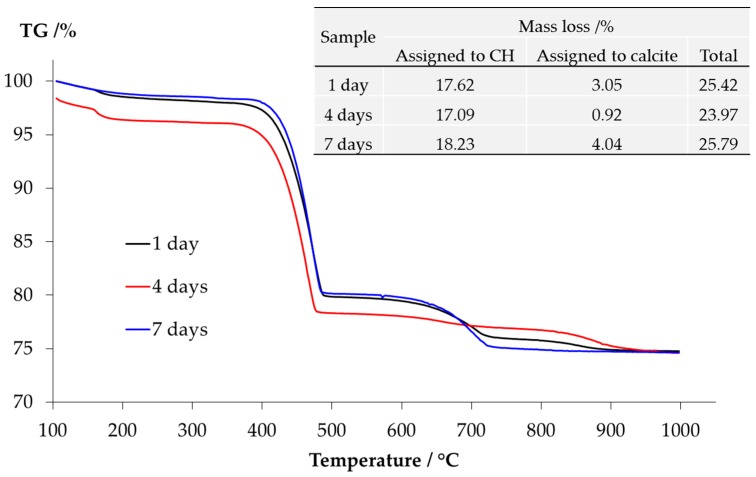
Mass loss of the sample from the middle layer determined by TGA.

The TGA results of the samples from the middle layer are shown in Figure 10. The content of Ca(OH)_2_ and calcite present in the middle layer can be calculated based on the mass loss. The method of calculation is provided elsewhere [16]. Apparently, the consistency among the results of the samples reacted for one, four and seven days confirms the trend observed by the XRD that, the storage period indeed had no significant influence on the kinds of phases in the middle layer. More particularly, the significant mass loss located between 400 °C and 500 °C verifies the abundant presence of Ca(OH)_2_. The mass loss due to calcite is also clear between 650 °C and 800 °C. However, the presence of calcium alkali silicate is uncertain since the curves have extremely low but non-negligible slopes ranging between 100 °C and 400 °C. The mass loss within this range can be attributed to the dehydration of calcium alkali silicate or alkali silicate [16].

**Figure 11:**
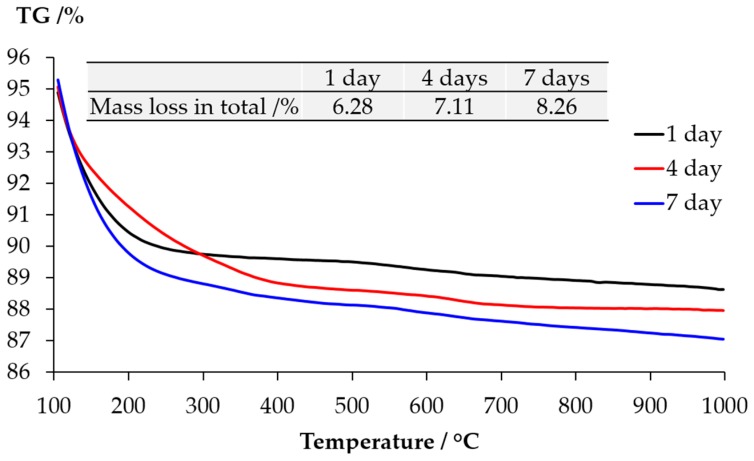
Mass loss of the sample from the slurry layer determined by TGA.

The TGA results of the samples collected from the slurry layer are shown as Figure 11. The disappearance of the typical mass lose contributed by either Ca(OH)_2_ or calcite confirms the XRD results that the slurry layer contained neither Ca(OH)_2_ nor calcite. Combing with the XRD results, the significant mass loss locates between 100 °C and 400 °C is due to the dehydration of alkali silicate rather than calcium alkali silicate since the typical broadening peaks from calcium alkali silicate were not found in the XRD results shown in Figure 9.

### 3.4. XRF Results

**Figure 12:**
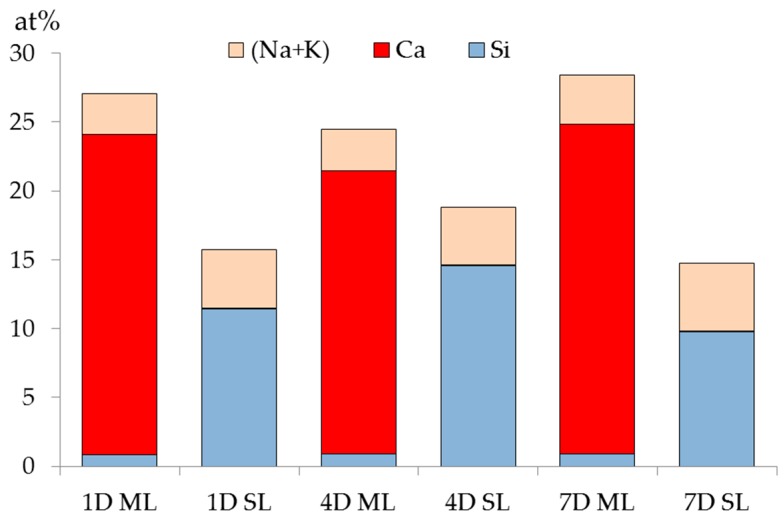
Mole fraction of calcium and silicon in the middle layer (ML) and the slurry layer (SL). For example, 1 D ML = the middle layer collected from the sample reacted for one day; 1 D SL = the slurry layer collected from the sample reacted for one day.

The XRF results of the samples from either the middle layer or the slurry layer with different reaction periods are summarized and plotted in Figure 12. Notably, the mole ratio of the elements including (O, Al, Fe, Mg and so on) are not given individually in this chart to avoid confusion.

Obviously, regardless of the storage period, calcium is abundant in the middle layer while rare in the slurry layer; and the silicon is rare in the middle layer while abundant in the slurry layer. Particularly in the middle layer, the mole fraction of silicon is no more than 1% with respect to that of calcium, which is more than 20%. The abundance of the calcium is consistent with the XRD and TGA results showing that portlandite and calcite were present in the middle layer. In addition, the content of alkalis (Na and K) is slightly higher in the slurry layer than in the middle layer regardless of the storage period. This is perhaps due to more alkalis were bonded by alkali silicate in the slurry layer. This also infers that the contents of alkalis present in the middle layer and the slurry layer are similar.

In the middle layer, where Ca(OH)_2_ is abundantly present, any silicate had to react with the excess Ca(OH)_2_ to form calcium alkali silicate, i.e., silicate cannot coexist with Ca(OH)_2_ in this region other than reacts with Ca(OH)_2_ to form calcium alkali silicate. Hence, the weak signal of silicon detected in the middle layer came from a trace amount of calcium alkali silicate. This confirms the suspicious presence of calcium alkali silicate, which was not detected by XRD and TGA in the middle layer due to its extremely low concentration.

In the slurry layer, regardless of the storage period, the mole fraction of calcium is lower than 0.1% while that of silicon is higher than 10%. This indicates that, on the one hand, no additional calcium arrived in the slurry layer considering the original mole fraction of calcium in the silica fume. On the other hand, silicon prevailed in the slurry layer, consistent with the XRD results shown in Figure 9 that the slurry layer was composed of unreacted silica fume or alkali silicate.

### 3.5. Existence of a Transport Barrier

Based on the XRD, TGA and XRF results mentioned in the last sections, some remarks need to be clarified before we continue. In the middle layer, the excess Ca(OH)_2_ present in this layer enabled the reaction between calcium and silicate to proceed as long as both of them were present in this area. However, the least amount of silicon detected by XRF as well as the evidence of excess portlandite present in this area suggests that, the reaction ceased due to a lack of sufficient reagent, i.e., silicate. Therefore, it is reasonable to believe that no additional silicate coming from the slurry layer arrived in the middle layer. The trace amount of silicate detected was perhaps from the dissolved silicate originally present in the liquid layer at the top, though the transport of trace amount of silicate cannot be absolutely excluded based on these results. However, this possible migration or supply of silicate was supposed to be limited, otherwise the excess Ca(OH)_2_ present in the middle layer can react with any silicate to form calcium alkali silicate resulting in an expected observation of the typical broadening peaks from calcium alkali silicate in the XRD results shown in Figure 8 as well as a higher silicon content in the XRF results shown in Figure 12. Hence, it is rational to conclude that the further migration of silicate from other positions, especially from the slurry layer beneath, to the middle layer was somehow prevented leading to an insufficient supply of silicate for its reaction with calcium in the middle layer. This implies that a transport barrier exists between the silicate source (the slurry layer) and the calcium source (the middle layer) to prevent the migration of silicate from the slurry layer to the middle layer against the great silicate concentration gradient. It should be noted here that, this transport barrier was indeed playing a similar role of the reaction rim as proposed by Ichikawa [10,11].

A similar situation happened in the slurry layer, a transport barrier was able to prevent the migration of calcium coming from other places, especially from the middle layer, to the slurry layer. Otherwise if any additional calcium was present in the slurry layer, it could react with abundant alkali silicate there to generate calcium alkali silicate resulting in an expected observation of the typical broadening peaks in the XRD results shown in Figure 9 as well as a higher calcium content in the XRF results shown in Figure 12.

With respect to alkalis, there are only slight differences between their concentrations in the middle layers and the slurry layers from different samples. This infers the migration of alkalis was not influenced by such a transport barrier.

Based on the above results and discussion, this transport barrier preventing the penetration of both calcium and silicate can only be located between the middle layer, which was the calcium source at the top, and the slurry layer, which was the silicate source at the bottom. Considering the layered appearance of this system as shown in Figure 7, the hard layer physically separated the middle layer and the slurry layer. Therefore, the hard layer was identified as the transport barrier preventing the migration of calcium and silicate. Accordingly, further emphasis will be cast on the hard layer itself to investigate its properties and explore its functioning mechanism as a transport barrier. It needs to be noted that, even though the transport barrier found in this study had comparable functions as the reaction rim, we will keep addressing it as transport barrier rather than reaction rim considering the simplified chemical model system used in this study.

### 3.6. BSE Imaging

**Figure 13:**
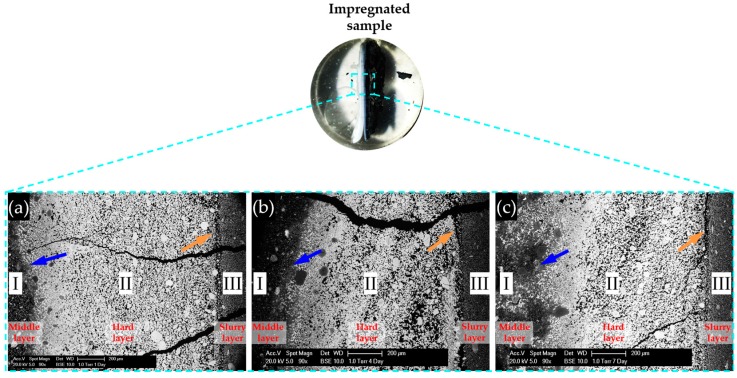
BSE images for the samples reacted for: (**a**) one day; (**b**) four days; and (**c**) seven days.

BSE images of samples with storage periods of one, four and seven days are shown in Figure 13a–c, respectively. The directions of the images are shown as well.

As shown in Figure 13, the BSE images of all the samples share some similarities. First, obviously, three regions with different grey levels can be found. According to the direction of the sample placement, these three regions can be assigned to the three phases found on the samples: adhesives from the middle layer on the upper surface of the hard layer; the hard layer itself; adhesives from the slurry layer on the lower surface of the hard layer. In particular, Region I was located at the upper surface of the hard layer belonging to the adhesives from the middle layer; Region II was the hard layer; and Region III was located at the lower surface of the hard layer belonging to the adhesives from the slurry layer. 

Obviously, compared to the other two regions, the region of the hard layer had the highest grey level indicating it was the most densified phase. The region of the adhesives from the slurry layer was relatively more uniform than the other two regions. The region with the adhesives from the middle layer contained fragmental particles with different size.

The border between the adhesives from the middle layer and the hard layer as indicated with the blue arrows in Figure 13, were not as clear as the ones between the hard layer and the adhesives from the slurry layer as indicated with orange arrows in Figure 13. In addition, several cracks can be noticed in all the BSE images in Figure 13. However, no products can be found in the interior of these cracks. This indicates the cracks did not form until the sample preparation. These cracks probably formed during the drying treatment.

### 3.7. EDS Results

**Figure 14:**
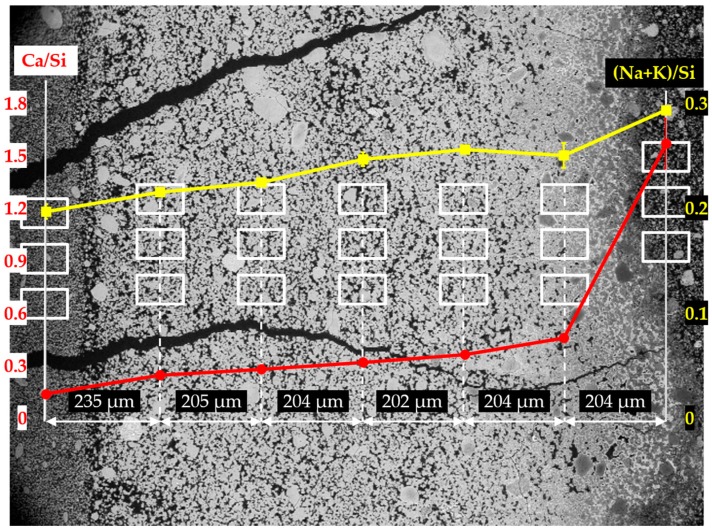
Changes of Ca/Si ratio and (Na+K)/Si ratio of the areas from the sample reacted for one day. The red curve represents the change of Ca/Si ratio, the yellow curve represents the change of (Na+K)/Si ratio.

**Figure 15:**
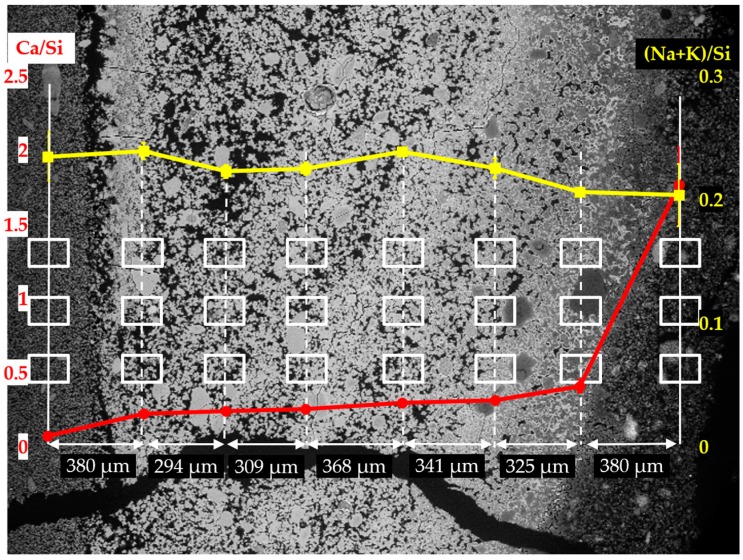
Changes of Ca/Si ratio and (Na+K)/Si ratio of the areas from the sample reacted for four days. The red curve represents the change of Ca/Si ratio, the yellow curve represents the change of (Na+K)/Si ratio.

**Figure 16:**
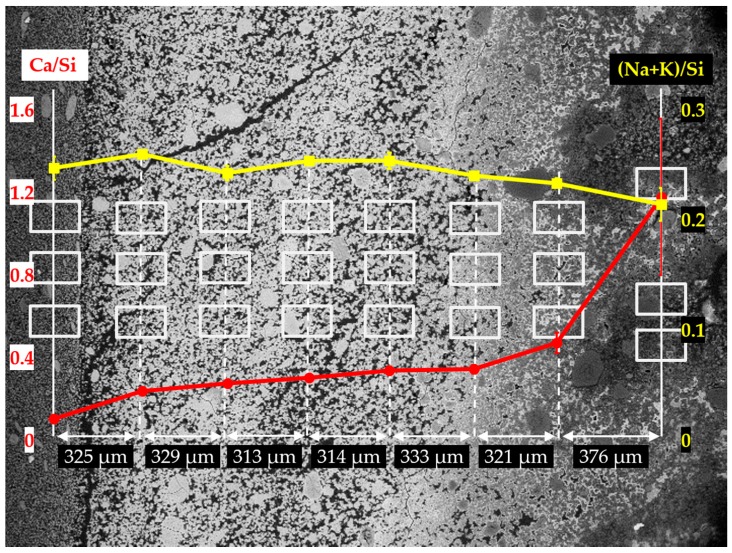
Changes of Ca/Si ratio and (Na+K)/Si ratio of the areas from the sample reacted for seven days. The red curve represents the change of Ca/Si ratio, the yellow curve represents the change of (Na+K)/Si ratio.

In order to investigate the elemental composition of the hard layer, EDS area-scanning was carried out on the selected areas of each sample. As shown in Figure 14, Figure 15 and Figure 16 for the sample reacted for one, four and seven days, respectively, the locations of the areas selected for EDS area-scanning are marked with rectangles. For the sake of convenience, the elemental composition of each area analyzed by EDS was expressed as Ca/Si ratio and alkali to silica mole ((Na+K)/Si) ratio. The averaged Ca/Si ratios and (Na+K)/Si ratios as well as their corresponding deviations of the areas from each column were plotted according to its relative position as indicated in the BSE images.

The EDS results of the sample reacted for one day are shown in Figure 14. Based on the results of Ca/Si ratio, two regions can be differentiated: the first region containing the six columns on the left having a low value of Ca/Si ratio; the second one containing the first column on the right having a high value of Ca/Si ratio. The Ca/Si ratios of the areas from the second region, in particular, were about 1.60 on the mean, while the Ca/Si ratios of the areas from the first region were lower than about 0.40.

Interestingly, the Ca/Si ratio dropped dramatically from about 1.60 to about 0.40 within a distance of 204 µm between the first and second columns from the right, confirming the existence of the border of the hard layer with the adhesives from the middle layer between the first and the second columns as marked with a blue arrow in Figure 13a. The huge decrease of the Ca/Si ratio across the border indicates that, the hard layer was able to keep large amount of calcium outside of its territory. It should be noted that, either the decreasing amount of calcium or the increasing amount of silicon present in these areas, can cause the decrease of Ca/Si ratio. However, for the situation here on the border of the hard layer with the adhesives from the middle layer where abundant Ca(OH)_2_ was present, the decreasing amount of calcium should be the dominant effect since the concentration of silicate in these areas was expected to be low. On the contrary, such a significant decrease of Ca/Si ratio was not found across the border of the hard layer with the adhesives from the slurry layer, as indicated with an orange arrow in Figure 13a. The Ca/Si ratio dropped from about 0.25 for the areas located on the hard layer to about 0.14 for the areas located on the adhesives from the slurry layer. This minor decrease was probably attributed to the abundant presence of silicate near the lower surface of the hard layer in contact with the slurry layer beneath. In other words, the relatively high concentration of silicate as well as the relatively low concentration of calcium limited the change of the Ca/Si ratio in this area.

Moreover, for the areas located within the territory of the hard layer, their Ca/Si ratio slightly decreased from about 0.45 to about 0.25 as getting away from the border of the hard layer with the adhesives in a distance of about 800 µm. This implies the hard layer was even capable of preventing migration of calcium and silicate within its territory. In addition, the changes of the (Na+K)/Si ratio had a smaller variation ranging from 0.20 to 0.30 indicating the weak interaction between the hard layer and the alkalis.

The EDS results of the analyzed areas of the sample reacted for four days are shown in Figure 15. Similar to the sample reacted for one day, two regions can be differentiated with respect to the values of Ca/Si ratio: the region containing the first column from the right with a high value of Ca/Si ratio; the region containing the other seven columns on the left with a low value of Ca/Si ratio. Based on the observation from the BSE image shown in Figure 13b, the region with high value of Ca/Si ratio was located on the adhesives from the middle layer; the region with the second column to the seventh column on the right was located on the hard layer itself; the first column on the left was located on the adhesives from the slurry layer.

Comparably, a dramatic decrease of the Ca/Si ratio was noticed from the first column on the right to the second one. The Ca/Si ratio dropped from about 1.80 to about 0.40 within a distance of 380 µm suggesting the existence of the border between the hard layer with the adhesives from the middle layer as indicated with a blue arrow in Figure 13b. Such a high value of Ca/Si ratio implies the presence of Ca(OH)_2_ in this region. Besides, the dramatic drop of Ca/Si ratio confirms the ability of the hard layer to stop the penetration of calcium into its matrix. With respect to the border of the hard layer with the adhesives from the slurry layer as indicated with an orange arrow in Figure 13b, the Ca/Si ratio dropped from about 0.24 to about 0.08 across the border. This decrease of Ca/Si ratio was mainly attributed to the significantly increasing concentration of silicate rather than the decreasing concentration of calcium confirming the hard layer was able to stop the transport of silicate as well. Moreover, the (Na+K)/Si ratio did not show significant changes across these regions consistent with the results of the sample reacted for one day.

In Figure 16, the EDS results of the sample reacted for seven days are given. Compared to the samples with the reaction periods of one day and four days, a similar trend of Ca/Si ratio variation across the borders has been found. A significant decrease of Ca/Si ratio was noticed across the border of the hard layer with the adhesives from the middle layer from about 1.20 to about 0.47 within a distance of 376 µm. Meanwhile, the Ca/Si ratio experienced another considerable decrease across the border of the hard layer with the adhesives from the slurry layer from about 0.25 to about 0.10. This is consistent with the results from the other two samples that, the hard layer was indeed able to prevent the transport of calcium and silicate across its borders. Furthermore, for the areas in the interior of the hard layer, their Ca/Si ratios slightly dropped from about 0.47 to about 0.25, suggesting the hard layer was also capable of preventing the migration of calcium and silicate in its interior. In addition, (Na+K)/Si ratio experienced a minor variation ranging from 0.21 to 0.25 consistent with the results of the other two samples.

**Figure 17:**
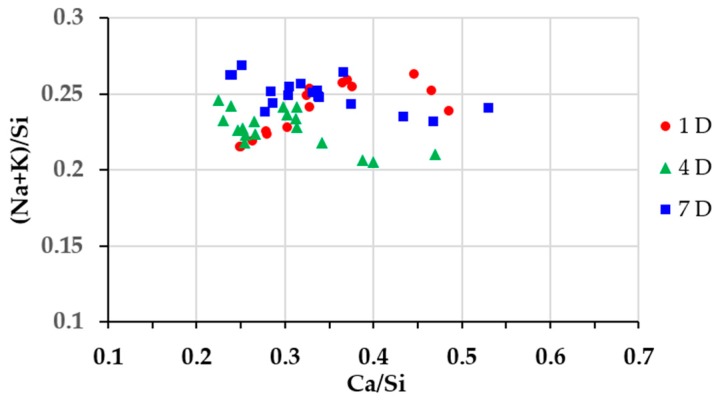
Elemental compositions of the analyzed areas on the hard layers from the samples with the storage period of one day (1 D), four days (4 D) and seven days (7 D).

With the help of the above results, the areas located at the hard layers itself from different samples can be identified. Thereafter, the Ca/Si ratio of these areas are plotted against their corresponding (Na+K)/Si ratio to reveal the elemental composition of the hard layer, as shown in Figure 17. The results appeared to be relatively concentrated implying the similarity among the samples with different storage periods. The Ca/Si ratio of calcium alkali silicate found on the hard layers was roughly ranging between 0.22 and 0.53; the (Na+K)/Si ratio was ranging between 0.20 and 0.26. This is different from the previous studies that the reaction rim found in real concrete was with either high Ca/Si ratio [7] or low Ca/Si ratio [9]. This is perhaps due to the relatively pure chemical model system used in this study compared to the heterogeneous conditions in real concrete. Moreover, the areas located closer to the lower surface of the hard layer which was in contact with the slurry layer, had a lower Ca/Si ratio; the ones located closer to the upper surface of the hard layer which was in contact with the middle layer, had a higher Ca/Si ratio. Interestingly, the Ca/Si ratios of all the areas located close to the lower surface of the hard layer in contact with the slurry layer were around 0.20. It seems this is the lowest Ca/Si ratio for the calcium alkali silicate to form the hard layer.

### 3.8. NMR Results

The powdered samples from the hard layer with different reaction periods were analyzed with ^29^Si NMR. For the sake of convenience, it is necessary to introduce the standard notation of Q^n^ nomenclature to interpret the NMR spectra [17]. Q represents a given tetrahedron; the exponent n represents the number of the associated oxygen with silicon. In this way, a Q^4^ tetrahedron has four bridging oxygen with silicon suggesting the existence of a complete interlinked silicon–oxygen organization, e.g., a 3D structure. A Q^3^ tetrahedron has three bridging oxygen and one non-bridging oxygen with silicon suggesting this tetrahedron has one bond available for bridging. Notably, the non-bridging oxygen can be bonded to another cation other than silicon, e.g., bonded to a proton to form an –OH group. A Q^2^ tetrahedron has two bridging oxygen and two non-bridging oxygen indicating the existence of a less cross-linked silicon–oxygen organization as compared to Q^3^. Q^2^ is usually found to be present as part of a chain. A Q^1^ tetrahedron has only one bridging oxygen with silicon and three non-bridging oxygen, which is usually found to be present at the end of a structure or as in a dimer. Q^0^ tetrahedron has four non-bridging oxygen usually present as monomer Si(OH)_4_. With the help of this nomenclature, the processes of polymerization and de-polymerization can be defined. The polymerization describes a process of interconnection between different tetrahedron with an increasing amount of Q^3^ or Q^4^ at the expense of Q^1^ or Q^2^. The de-polymerization describes an opposite process to the polymerization that the amount of Q^1^ or Q^2^ increases at the expense of Q^3^ or Q^4^.

**Figure 18:**
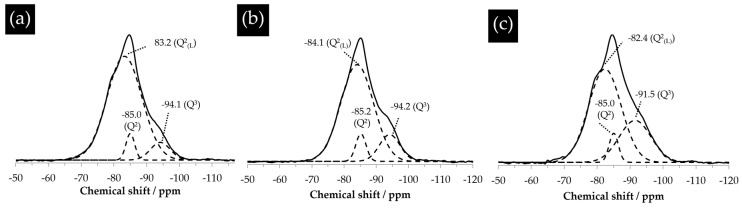
^29^Si nuclear magnetic resonance (NMR) spectra of the hard layer in the system reacted for: (**a**) one day; (**b**) four days; and (**c**) seven days. The solid lines are the experimental spectra, the dashed lines are the deconvoluted spectra.

The assignment of the peaks shown in Figure 18 was obtained by deconvolution. As shown in Figure 18, the powdered samples of the hard layers from different systems were mainly composed of Q^2^ with a chemical shift ranging between −79 ppm and −85 ppm and Q^3^ with a chemical shift ranging between −91 ppm and −98 ppm. The absence of the signal from Q^4^ in these spectra suggests there was no unreacted silica present in the hard layers. For the sample reacted for one day (Figure 18a), aside from the typical peaks of Q^2^ at −85 ppm and Q^3^ at −94.1 ppm, a shoulder centered at −83.2 ppm was found. This was attributed to the altered environment of Q^2^ by its linkage with a proton and so presence as a bridging tetrahedron [18]. This altered Q^2^ is named Q^2^_(L)_ here and later on. Similarly, for the samples reacted for four days and seven days, the resonance of Q^2^ slightly shifted towards the downfield, leaving a minor shoulder next to the main peak of Q^2^.

Based on the deconvoluted spectra shown in Figure 18, the fraction of each kind of silica tetrahedron can be obtained according to the percentage of the area under its deconvoluted spectrum. As shown in Table 2, the fraction of Q^2^_(L)_ decreased and that of Q^3^ increased with the increase of the reaction period. In other words, the polymerization degree of the calcium alkali silicate from the hard layer increased with the increase of the storage period. In the meantime, however, the fraction of Q^2^ experienced an increase from one day to four days and a decrease from four days to seven days. It should be noted that the observed increase of Q^3^ and decrease of Q^2^_(L)_ with the storage period does not necessarily mean that Q^3^ was formed at the expense of Q^2^_(L)_ as usual. This polymerization can also be caused by the interlinkage of the alkali silicate externally from the slurry layer with the calcium alkali silicate in the hard layer near its lower surface [19]. Meanwhile, on the contrary, the interaction between Ca(OH)_2_ present in the middle layer and the calcium alkali silicate in the hard layer near its upper surface would unavoidably cause the de-polymerization of the calcium alkali silicate [20,21], given the previous results that the content of calcium increased near the upper surface of the hard layer. Therefore, the polymerization degree of the hard layers was determined by polymerization and de-polymerization happening on either surface of the hard layer.

Therefore, an assumption can be made based on the results shown in Table 2 and given as follows. Based on the increasing amount of Q^3^, the polymerization dominated the de-polymerization in the system. The rate of the de-polymerization caused by the interaction of calcium with calcium alkali silicate near the upper surface of the hard layer should be much lower than that of the polymerization happening near the lower surface of the hard layer.

### 3.9. Self-Thickening Process and Evolution of the Transport Barrier

**Figure 19:**
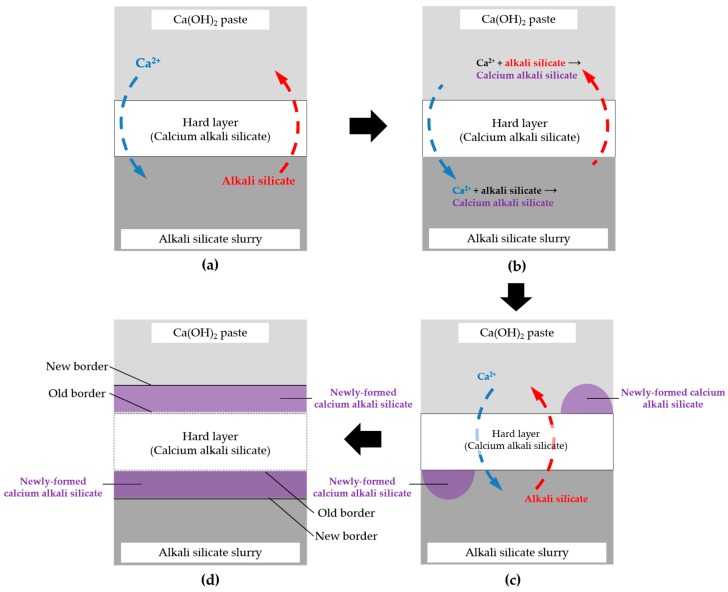
Schematic representation of the self-thickening process: (**a**) migration of Ca^2+^ and alkali silicate through the hard layer; (**b**) interaction of Ca^2+^ with alkali silicate on the either side of the hard layer after the migration; (**c**) formation and accumulation of calcium alkali silicate on either side of the hard layer; and (**d**) extension of the borders of the hard layer by the accumulation of calcium alkali silicate.

Based on the above results and discussion, the evolution mode of the hard layer as a transport barrier realized by a self-thickening process can be visualized by a thought experiment shown schematically in Figure 19 and illustrated in detail as follows.

If any free calcium with a considerable amount is present on the lower “temporary” surface of the hard layer, which is in contact with the slurry layer by the migration through the hard layer, it can immediately react with the abundant alkali silicate present there to form calcium alkali silicate leading to the thickening of the hard layer until the concentration of calcium is too low to trigger this interaction. For example, in this study, such a threshold of Ca/Si ratio for enabling the formation of the hard layer is about 0.20 according to the EDS results of the areas located at the border of the hard layer with the slurry layer. Alternatively, if any alkali silicate is present on the upper “temporary” surface of the hard layer which is in contact with the middle layer, it can react with abundant Ca(OH)_2_ to form calcium alkali silicate leading to the thickening of the hard layer until the amount of silicate is too low to trigger this interaction as well. This self-thickening process, as a result, enables the growth of the hard layer towards either the middle layer for the upper surface at the top or the slurry layer for the lower surface at the bottom. In other words, the migration of calcium and silicate is completely prevented as soon as this process stops. Moreover, if any new transport path of calcium and silicate such as a micro crack is generated across the hard layer, the interaction mentioned above can “heal” this “wound” until the path is fully blocked.

### 3.10. Further Discussion

Combining all these results, it is possible to depict an image of all what is happening on the interface between alkali silicate and calcium in a calcium–alkali–silicate system. Based on this, further understanding about the formation and evolution of the reaction rim can be provided.

(1) The formation of alkali silicate: after the reactive silica meets the NaOH solution, the silica tetrahedral networks are destroyed to form alkali silicate.
(2) The formation of calcium alkali silicate: alkali silicate can interlink with calcium to form calcium alkali silicate once they meet with each other.
(3) The accumulation of calcium alkali silicate: the formation of calcium alkali silicate will continue as long as both alkali silicate and calcium are present.
(4) The formation of the transport barrier: the accumulation of calcium alkali silicate near the interface between alkali silicate and Ca(OH)_2_ physically separates these two phases.
(5) The thickening and evolution of the transport barrier: as long as additional calcium or silicate can penetrate the interface which is composed of the previously formed calcium alkali silicate to reach the other reactant to react with, this barrier can be thickened by the newly formed calcium alkali silicate.
(6) Once calcium or silicate exhausts near the interface of the transport barrier either with alkali silicate (e.g., the lower surface of the hard layer in this study) or with Ca(OH)_2_ (e.g., the upper surface of the hard layer in this study), the isolation of alkali silicate and Ca(OH)_2_ is reached. Thereafter, the transport of calcium and silicate are prevented.
(7) Whenever a disturbance opens a new path (e.g., micro cracks) to let alkali silicate or calcium pass through, additional interaction between alkali silicate and calcium will be triggered again until the products “heal” the breakage.

As a consequence, depletion of Ca^2+^, further polymerization of alkali silicate and even the accumulation of alkali silicate due to the continuous interaction of reactive silica with the alkaline solution, as reported by other researches [12,22,23], are likely to be realized in the region of the slurry layer. Once the interior expansive force exceeds the strength of the transport barrier, typical deterioration due to ASR occurs.

## 4. Conclusions

In this work, we simulated the chemical interaction of reactive silica, alkaline solution and calcium in an original simplified calcium–alkali–silicate system. The emphasis was led on the interaction between alkali silicate and Ca(OH)_2_ to provide information and inspiration about the formation and evolution of the reaction rim in real concrete from a fundamental chemical point of view.

A transport barrier located on the interface between alkali silicate and Ca(OH)_2_ was found. It separated these two phases and prevented the transport of both calcium and silicate through itself leading to a layered system. This transport barrier was composed of calcium alkali silicate with a Ca/Si ratio ranging from 0.22 to 0.53 and a (Na+K)/Si ratio ranging from 0.20 to 0.26. In addition, the calcium alkali silicate from the transport barrier was mainly composed of the silicon–oxygen organization of Q^2^ and Q^3^, its polymerization degree increases with the storage period.

## Figures and Tables

**Table 1:** Chemical composition of the used silica fume (wt. %).

Compositions	SiO_2_	CaO	Al_2_O_3_	Fe_2_O_3_	MgO	Na_2_O	K_2_O	SO_3_
Content	94.2	0.6	1.0	0.5	0.7	1.0	1.1	0.3

**Table 2:** Area percentages of the deconvoluted components in the ^29^Si nuclear magnetic resonance (NMR) spectra.

Storage Time	Q^2^_(L)_	Q^2^	Q^3^
	(%)		
One day	86.2	5.5	8.3
Four days	79.2	7.1	13.7
Seven days	65.4	5.8	28.8
